# Ultrasound-Assisted Extraction Followed by Inductively Coupled Plasma Mass Spectrometry and Multivariate Profiling of Rare Earth Elements in Coffee

**DOI:** 10.3390/foods14020275

**Published:** 2025-01-16

**Authors:** Aleksandra Savić, Jelena Mutić, Milica Lučić, Jelena Vesković, Andrijana Miletić, Antonije Onjia

**Affiliations:** 1 Anahem Laboratory, Mocartova 10, 11160 Belgrade, Serbia; 2 Department of Analytical Chemistry, Faculty of Chemistry, University of Belgrade, 11158 Belgrade, Serbia; 3 Innovation Center of the Faculty of Technology and Metallurgy, 11120 Belgrade, Serbia; 4 Faculty of Technology and Metallurgy, University of Belgrade, 11120 Belgrade, Serbia

**Keywords:** Arabica, Canephora, surrogate, REEs, inductively coupled plasma mass spectrometry, positive matrix factorization

## Abstract

A rapid and efficient ultrasound-assisted extraction (UAE) procedure followed by inductively coupled plasma mass spectrometry (ICP-MS) was developed for the determination of 14 rare earth elements (REEs) (La, Ce, Pr, Nd, Sm, Eu, Gd, Tb, Dy, Ho, Er, Tm, Yb, Lu), along with yttrium (Y) and scandium (Sc), in coffee samples. The method was validated using certified reference material (NIST SRM 1547), recovery tests at four fortification levels, and comparisons with microwave-assisted digestion (MAD). Excellent accuracy and precision were achieved, with recovery rates ranging from 80.1% to 112% and relative standard deviations (RSD%) below 14%. Limits of detection (LODs) ranged from 0.2 ng/kg (Yb) to 0.16 µg/kg (Nd). Total REE concentrations varied between 8.3 µg/kg and 1.1 mg/kg, with the highest individual mean concentrations (µg/kg) observed for Ce (11.7), La (6.0), and Sc (4.7). The lowest individual mean concentrations (µg/kg) were for Ho (0.16), Lu (0.066), and Tm (0.063). Multivariate analysis of REE profiles from 92 coffee samples collected in Serbia revealed clear distinctions between ground roasted and instant coffees, as well as between different surrogate blends. This study indicated that the determination of coffee’s geographical origin was not possible due to the diverse types, blends, and additives. However, differences in REE profiles suggest potential classification based on variety. REEs pose a negligible health risk to coffee consumers, with HI values ranging from 4.7 × 10^−8^ to 6.3 × 10^−6^ and TCR ranging from 2.6 × 10^−14^ to 3.5 × 10^−12^.

## 1. Introduction

Coffee most likely originated from the northern regions of Africa, and today, it is commercially cultivated in approximately 70 countries worldwide. These countries span Central and South America, the Caribbean, Africa, and Asia [1,2,3]. The consumption and economic cultivation of coffee crops began in the 15th century [1,4]. Generally, two main species of coffee plants are grown: Arabica (*Coffea arabica*), which has a lower caffeine content and accounts for 70% of all coffee production, and Robusta (*Coffea canephora*), which has a significantly higher caffeine content and accounts for 30% of all coffee production [3,5]. The chemical profile and quality of coffee (including flavor, taste, aroma, and style) are influenced by several factors, including the coffee species, soil, field practices, climate, and processing methodology (roasting) [6,7,8].

Rare earth elements (REEs) include 17 elements that encompass 15 lanthanoids, as well as scandium (Sc), and yttrium (Y). The lanthanide series (from Z = 57 to Z = 71) includes the following elements: lanthanum (La), cerium (Ce), praseodymium (Pr), neodymium (Nd), promethium (Pm), samarium (Sm), europium (Eu), gadolinium (Gd), terbium (Tb), dysprosium (Dy), holmium (Ho), erbium (Er), thulium (Tm), ytterbium (Yb), and lutetium (Lu) [9,10]. These elements are non-essential to living organisms and have low to moderate toxicity [11,12]. REEs are usually classified as light REEs (LRREs) from La to Sm and heavy REEs (HREEs) from Eu to Lu, including Y [13,14]. Scandium is not classified into any sub-group because of its small ionic radius [15,16].

REEs have applications in different industries, as well as in medicine and agriculture. They are used not only for producing high-tech products but also in traditional industries. In agriculture, these elements are also used as fertilizers, plant growth promoters, and feed additives for livestock, poultry, and aquaculture [9,12,16]. In nature, they occur in the form of minerals, and 95% of these elements are found in bastnasite, monazite, and xenotime [10]. Under natural conditions, the availability of REEs in the environment (soil [17], water [18], atmosphere [19], and plants [20]) is low. However, REE mining processes and rapidly growing REE usage in modern industry and everyday life can increase REE concentrations and pose a threat to environmental and human health [9,21,22].

Most studies have shown that REEs are present at low concentrations in most food products [23,24]. The chemical composition of food is directly influenced by the soil in which it is grown. Therefore, REE content can serve as a means of authenticating various food products. In previous research, REEs have provided information on the geographic origins of wine [25], lentils [26], honey [27], and *Ruditapes philippinarum* (clam) [28]. To trace the origins of green and roasted coffee, several studies have investigated the content of REEs alongside other elements [3,29,30].

Concentrations of REEs in soils, water, and food are usually in traces, so analyses of these elements in food matrices are usually performed using inductively coupled plasma mass spectrometry (ICP-MS). This analytical technique is most commonly used for determining REEs in biological samples, including various foodstuffs, such as tomato plants [31], olive oils [32], meat and dairy products [33], pistachios [34], coffee [29,35], etc. Among the analytical methods for authenticating the geographical origin of food, ICP-MS stands out as a leading technique due to its robustness, accuracy, and sensitivity in detecting inorganic elements in food samples [36]. In general, food samples are usually prepared by acid digestion using concentrated acids. This type of sample preparation has several drawbacks, such as reducing sensitivity caused by a large volume dilution before instrumental analysis, and it can also influence the precision of element determination due to possible contamination. Ultrasound-assisted extraction (UAE) is a promising alternative to microwave-assisted digestion (MAD). The primary advantages of the UAE method are shorter sample preparation time (extraction), the use of diluted acids (which reduce waste and eliminate the need for dilution), and the application of lower temperatures and pressures during extraction. All these factors contribute to lower costs, increased safety, simplified sample preparation, and give more efficient and cleaner method [37,38,39]. Lima et al. (2000) compared UAE and MAD for the determination of Cd, Cu, and Pb in biological and sediment samples, finding that UAE required less solvent and energy, aligning with green chemistry principles [40]. Furthermore, Fotouh R. Mansour et al. (2024) utilized the Modified GAPI (MoGAPI) tool to evaluate the greenness of several analytical methods, including UAE and MAD methods [41]. Their findings indicated that the UAE method generally scores higher on greenness metrics such as the Analytical Eco-Scale (AES) and Analytical GREEnness Metric (AGREE) due to its lower solvent consumption and reduced waste generation. Although specific studies applying green metric scales to UAE and MAD are limited, the general principles suggest that methods requiring fewer hazardous reagents and less energy consumption score higher on green metric scales. The main novelty of this study lies in the application of UAE, in combination with ICP-MS, for determining REEs in various types of coffee.

The objective of the present study was to develop an accurate and precise method for determining REEs in commercial ground roasted and instant coffee. Additionally, the study aimed to assess the REE profiles of coffee originating from different regions worldwide. A total of 92 coffee samples were analyzed using two sample preparation procedures (MAD and UAE). These procedures were validated using certified reference material (CRM). To the best of our knowledge, this study is the first to investigate the REE concentrations in coffee samples by combining UAE and ICP-MS analysis and to assess REE patterns using multivariate methods.

## 2. Materials and Methods

### 2.1. Samples and Reagents

The samples of various coffees and coffee surrogates (*n* = 92) were obtained from the local market in Belgrade, Serbia, in May 2024. Samples included 37 ground roasted coffees, 12 ground roasted coffees in capsules, 24 instant coffees with additives, 15 instant coffees, and 4 coffee surrogates. A detailed description of the analyzed samples is provided in Supplementary Materials Table S1.

All reagents used for digesting coffee samples were of analytical reagent grade. Hydrogen peroxide (30%, *w*/*w*) was provided by Lachner (Neratovice, Czech Republic), and nitric acid (67–70%, *v*/*v*) was supplied by Fisher Chemical (Merelbeke, Belgium). All solutions were prepared and diluted using Milli-Q water (Millipore, Bedford, MA, USA) with a resistivity of 18.2 MΩ·cm. Internal standard (Rhodium, Rh) and multi-element solutions (10 mg/L each) were purchased from AccuStandard (New Haven, CN, USA). All glassware and polypropylene tubes used in the analysis were soaked in 10% HNO_3_ overnight, rinsed with ultrapure water, and dried before use.

### 2.2. Samples Digestion and Instrument Analysis

In this study, coffee samples were prepared using two methods for element analysis: the conventional one, MAD, and the alternative one, UAE.

For MAD, a mass of 0.250 g was weighed and transferred into a polytetrafluoroethylene (PTFE) tube. This step was followed by the addition of 4 mL of 67–70% HNO_3_ and 1 mL of 30% H_2_O_2_. The tubes containing the samples were then subjected to a digestion process using a microwave oven MARS 5 (CEM, Matthews, NC, USA). The digestion regime was 25 min to 120 psi and held for 10 min (operating parameters were power 1600 W, pressure sensor control). After cooling, the mixtures were added to a final volume of 25 mL ultrapure water. The solutions were centrifuged, diluted five times, and measured at ICP-MS.

The UAE procedure was performed following the method of Gohlke et al. (2024) [42] with modifications. A precise mass of 0.250 g was weighed and transferred to a 15 mL Falcon polypropylene (PP) tube featuring screw caps (Biosigma S.p.A, Cona, Italy). Then, 10 mL of the extraction solution (1 M HNO_3_) was added. Prior to the sonication, the sample was vortexed for 1 min; afterward, sonication was conducted for 15 min in an ultrasonic bath (Elmasonic S15H, Elma Schmidbauer GmbH, Singen, Germany) operating at 95 W of effective ultrasonic power, a frequency of 37 kHz, and a temperature of 80 °C. The resulting mixtures were then centrifuged at 3000 rpm for 3 min. The obtained supernatants were diluted with ultrapure water (18.2 MΩ·cm) to 25 mL in a polymethyl-pentene (PMP) volumetric flask (class A, VITLAB GmbH, Großostheim, Germany). The solutions were stored at 4 °C until analysis.

The analysis of 14 REEs (La, Ce, Pr, Nd, Sm, Eu, Gd, Tb, Dy, Ho, Er, Tm, Yb, and Lu), Y, and Sc was performed using an inductively coupled plasma mass spectrometer (Thermo Scientific iCAP TQ ICP-MS, Bremen, Germany. The correction for interferences caused by polyatomic ions and other unwanted species was made using the KED (Kinetic Energy Discrimination) mode for all elements. The instrument operating parameters are presented in Table 1.

All coffee samples and NIST SRM 1547 were prepared and analyzed in their original state, without prior drying. The moisture content was subsequently determined (oven-based drying at 103 °C until a constant weight was reached, as described in ISO 11294 [43]), and results were expressed on a dry weight basis. Moisture content ranged from 1.1% to 4.3% for roasted ground coffees and from 1.2% to 4.7% for instant coffees.

### 2.3. Analytical Method Validation

External standard calibration combined with an internal standard (Rh) was employed to mitigate matrix effects and compensate for instrumental drift. Just to note that in this work, internal standard calibration was not used. Instead, the internal standard served to trigger recalibration of the ICP-MS instrument when a significant deviation in the internal standard signal occurred, below 80% or above 120% of the initial value. The calibration standards were prepared using the multi-element standard solution (PE-MECAL2-ASL-1, Accu Standard, New Heaven, CN, USA) and simulated matrix solutions that represent simulated digestions, containing element concentrations typical of those found in coffee as a matrix. A matrix solution containing Ca (1000 mg/L), Mg (1500 mg/L), K (15,000 mg/L), Na (300 mg/L), Cl (1400 mg/L), C (3.3%) as acetic acid, P (300 mg/L), S (200 mg/L), and F (200 mg/L). An internal standard was then added to reach a final concentration of 10 µg/L of Rh [7,44]. The method’s accuracy was confirmed by analyzing the CRM of botanical material obtained from the National Institute of Standards and Technology (NIST, Gaithersburg, MD, USA): Standard Reference Material 1547 (NIST SRM 1547) Peach Leaves. Analyses and measurements of all samples and CRM were carried out in triplicate. Additionally, the recovery of REEs was determined using four levels of fortification (0.5; 5.0; 40, and 200 µg/kg). Specifically, two coffee samples (one ground roasted coffee and one instant coffee) were spiked after digestion. Three separate subsamples of the same coffee were spiked and analyzed three times. The homogeneity of the spiked samples was ensured by vortexing for 1 min. The indicative values of the REE concentrations were obtained by analyzing the coffee samples ten times. The limit of detection (LOD) and limit of quantification (LOQ) were calculated as three and ten times the standard deviation (SD) of six measurements of analytical blank divided by the slope of the calibration curve.

### 2.4. Multivariate Statistics

Several multivariate statistical methods were employed to analyze the data. Pearson correlation analysis, principal component analysis (PCA), and hierarchical cluster analysis (HCA) utilizing the R software package, version 4.1.2 were performed to assess the linear relationships between REEs, to reduce dimensionality while preserving the variability within the data, and to classify the coffee samples into clusters based on similarity, allowing the grouping of REEs with shared characteristics. Additionally, the positive matrix factorization (PMF) method, using the EPA PMF version 5.0 software, was employed to decompose the data matrix into source profiles and contributions, providing insights into the underlying factors influencing the REE content in coffee samples.

### 2.5. Health Risks from REE in Coffee

The potential adverse human health effects of REEs present in coffee for consumers were evaluated using the Human Health Risk Assessment (HRA) model developed by the United States Environmental Protection Agency (USEPA) [45]. The model estimates the hazard index (HI) and target cancer risk (TCR) for assessing health risks. All model equations and exposure factor values used in the model are presented in Tables S2 and S3. The reference dose (RfD) and cancer slope factor (CSF) for the assessed REEs were set at 0.02 mg·kg^−1^·bw·day^−1^ and 3.2 × 10^−12^ kg·bw·day·mg^−1^ [46].

## 3. Results and Discussion

### 3.1. Method Development and Validation

The limits of detection (LODs), correlation coefficient (R^2^), and calibration equations are presented in Table 2. The linearity of the calibration curves was satisfactory for all elements, with an R^2^ value above 0.995. The five point calibration curves covered the concentration range from 1 to 25 µg/L. LOD values were in the range of 0.002 µg/kg (Yb) to 0.41 µg/kg (Sc).

**Figure 1:**
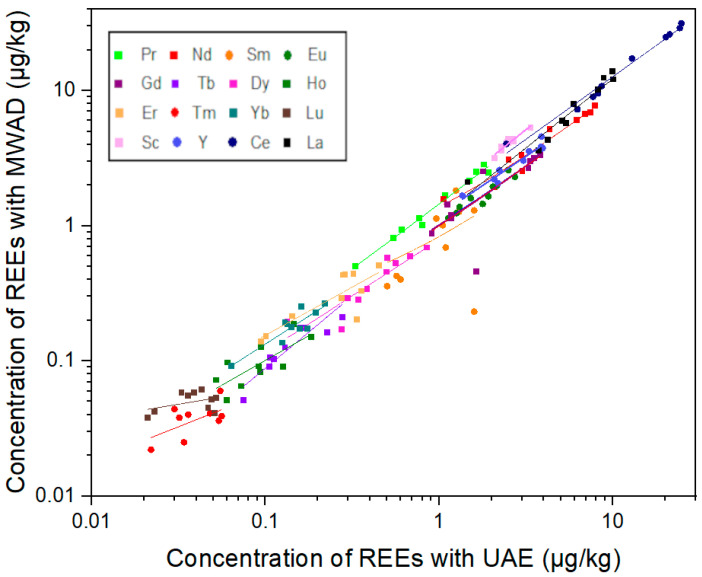
Linear regressions between REE concentrations in coffee after MAD and UAE (*n* = 10).

Microwave-assisted digestion methods are widely used to extract elements from various foodstuffs [47,48,49]. This method has previously been used by other researchers for the digestion of coffee samples to analyze REEs [3,29,30]. However, these methods often require a large amount of reagents, which can affect the measurement of elements present at low concentrations [37,50]. Ultrasound-assisted extraction offers a promising alternative with several advantages over conventional acid digestion methods, including reduced extraction time, the use of diluted acids (resulting in less waste and improved safety), simplicity, and lower operating costs [38,51]. Ultrasound waves enhance the extraction process via acoustic cavitation, which induces rapid and localized fluctuations in temperature and pressure, resulting in cell wall rupture [52]. In this context, we developed a UAE-based method for extracting REEs from coffee samples. To validate this method, a comparison of the UAE with MAD was performed, and a CRM was analyzed. The linear regressions between REE concentrations in MAD and UAE coffee samples (*n* = 10) are shown in Figure 1, while the measured concentrations are presented in Supplementary Materials Table S4. The results showed that the UAE procedure was an efficient and valid alternative to MAD, except for Sc and Ce, which had slightly lower extraction efficiency using the UAE procedure.

Figure 1 presents a linear regression analysis of REEs in coffee samples. The plot compares the concentration of REEs obtained using two different methods: MAD and UAE. The results of the linear regression analysis reveal varying degrees of correlation (Table S5) between concentrations. High correlation coefficients (R^2^ > 0.9) were obtained for Pr, Nd, Sc, Y, Ce, and La, indicating strong predictability and reliability of the method. Moderate to strong correlations (0.7 ≤ R^2^ ≤ 0.9) were observed for Eu, Gd, Tb, Dy, and Yb, suggesting that while the UAE method is fairly reliable, some variability remains unexplained. Lower correlations (R^2^ < 0.7) were found for Ho, Er, Tm, Sm, and Lu, indicating weaker predictability, particularly in cases where the element concentrations were very low. These findings suggest that while UAE is a suitable alternative to MAD for most REEs, further refinement may be required for elements with low concentration levels to enhance the accuracy and reliability of the method.

The accuracy of the measurements was verified by preparing and analyzing the CRM in the same way as the coffee samples. Although NIST SRM 1547 is not coffee-specific, it provides a plant-based matrix similar to coffee. The CRM data are presented in Table S6. The percentage recovery was obtained in the range of 93.5–112%. The precision of the method was determined by the relative standard deviation (RSD), which ranged from 0.63% to 11.9%. The method validation showed that the obtained concentrations of Eu, Gd, Nd, Sm, Sc, Tb, and Yb were not significantly different from the CRM values, based on the 95% confidence intervals (CIs, Table S6). Significant differences were observed for Ce and La. Additionally, the accuracy of the method is confirmed by spiking experiments. The initial concentrations of spiked coffee samples (ground roasted coffee and instant coffee) are presented in Table S7. The recoveries of spiked coffee samples ranged from 80.1 to 108% for UAE experiments, while those for MAD experiments ranged from 79.3 to 113%. The relative standard deviation (RSD%) of spiking experiments ranged from 1.8 to 13.0% for UAE experiments and from 1.5 to 12.3% for MAD experiments (Supplementary Materials Table S8a–d).

### 3.2. REE Content in Coffee

**Figure 2:**
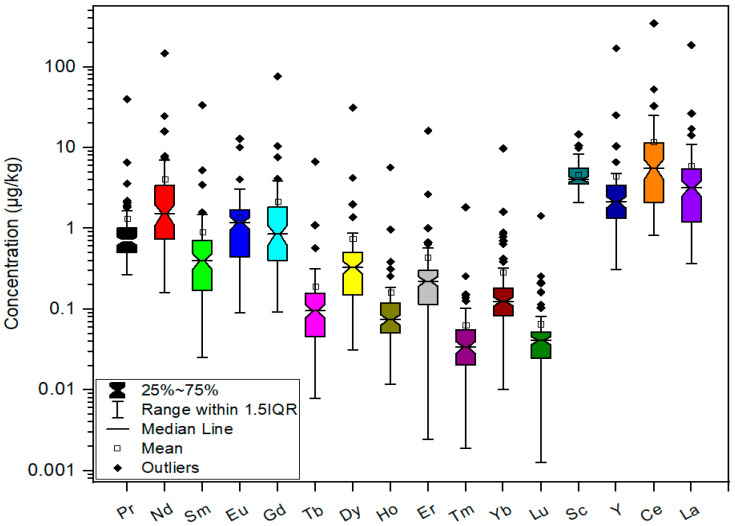
Box plots of REE concentrations in analyzed coffee samples.

The descriptive statistics for REEs in the studied coffee samples are presented in Figure 2 and Supplementary Table S9. Box plots were used to display the distribution of REEs, with concentrations presented on a logarithmic scale.

The total REE content ranged from 8.35 to 1097 µg/kg. The highest total content was measured in the surrogate sample (100% chicory). Other samples containing surrogates also exhibited higher total REE contents than the pure coffee samples. The lowest REE content was observed in instant coffee with additives (Cappuccino Irish Cream). Generally, instant coffees and instant coffees with additives had lower total REE contents than other analyzed samples. Two samples from Rwanda and Peru had significantly lower total REE content than other ground roasted coffees. This indicates that geographical origin may influence the REE content.

The mean concentrations of REEs followed the order Ce (11.7 µg/kg) > La (5.96 µg/kg) > Sc (4.68 µg/kg) > Y (4.43 µg/kg) > Nd (4.11 µg/kg) > Gd (2.45 µg/kg) > Pr (1.33 µg/kg) > Eu (1.28 µg/kg) > Sm (1.09 µg/kg) > Dy (0.740 µg/kg) > Er (0.438 µg/kg) > Yb (0.286 µg/kg) > Tb (0.191 µg/kg) > Ho (0.161 µg/kg) > Lu (0.066 µg/kg) > Tm (0.063 µg/kg). REEs (Tb, Sm, Ho, Er, Dy, Tm, and Lu) were below the corresponding LOD values in 23 coffee samples (3 ground roasted, 18 instant, and 2 ground roasted coffees in capsules). The minimum and maximum concentrations of REEs align with the concentrations reported by Vezzulli et al. (2023) [30], except for the maximum concentration of Eu, which was higher in our study (Table 3). The maximum concentrations of Eu are in good agreement with the results of Barbosa et al. (2014) [29] and Messaoudi et al. (2018) [53]. On the other hand, the maximum Ce and La concentrations reported for coffee samples from India [30] and Brazil [35] were significantly higher than the maximum values obtained for our coffee samples. Our results for pure coffee samples agree with those of Santato et al. (2012) [3] for 62 raw green coffee samples originating from Central America, South America, Africa, and Asia. Compared with pure coffee samples, surrogates and coffee with surrogates (2% chickpeas) exhibited higher minimum and maximum values for all REEs. For all elements, skewness and kurtosis values were greater than 2, indicating that the distribution of data was not normal; the data were highly skewed to the right and too peaked. Before further analyses of the normalized data, the logarithm transformation was applied.

### 3.3. Multivariate Analyses

**Figure 3:**
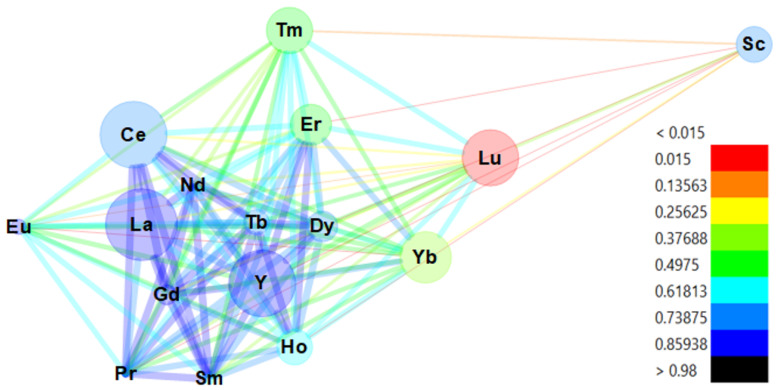
Pearson correlation network among concentrations of 16 different REEs in coffee samples (*n* = 92). Only positive correlations are represented: blue lines indicate strong positive correlations, light blue lines indicate moderate correlations, and the red line indicates weak positive correlations.

Pearson correlation analysis was employed to investigate the relationships among the analyzed REEs (Figure 3, Supplementary Materials Table S10). Positive correlation coefficients indicate a positive relationship, whereas negative values signify a negative relationship. Typically, a strong correlation exists between two parameters if the coefficient falls within the range of 0.7 to 1.0, a moderate correlation between 0.5 and 0.7, and a weak correlation if the coefficient is less than 0.5. Most REEs exhibited positive correlations, except for Sc with Nd (r = −0.247), Sc with Sm (r = −0.172), Sc with Eu (r = −0.218), Sc with Tb (r = −0.036), Sc with Ce (r = −0.260), and Sc with La (r = −0.266). The correlation coefficient of Sc with other elements indicated weak positive correlations. Additionally, Eu, Lu, Tm, and Yb generally exhibited moderate or weak correlations with other elements, suggesting that they originated from sources different than the rest of the REEs. However, all other REEs showed strong (or closely to strong) correlations with each other.

**Figure 4:**
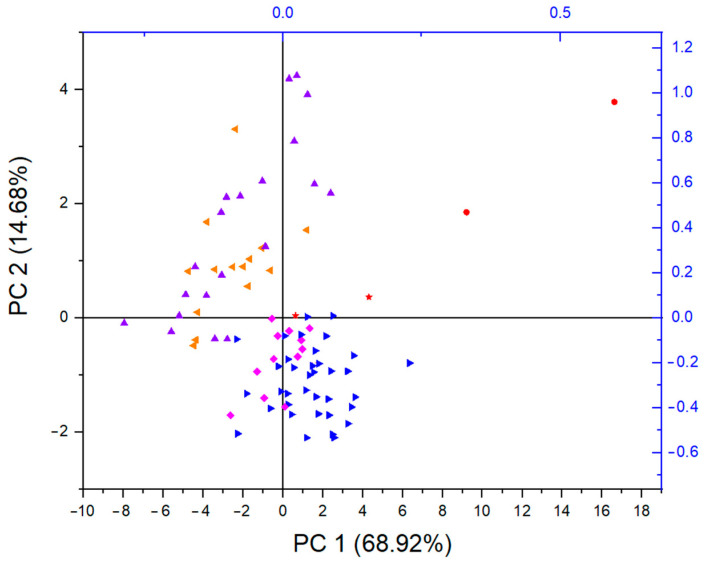
Principal component analysis (PCA). Score plot for 92 coffee samples based on rare earth element (REE) concentrations: blue ►—ground roasted coffees; orange ◄—instant coffees; violet ▲—instant coffees with additives; pink ♦—ground roasted coffees in capsules; red ●—surrogates for coffee; red star—coffees with surrogates (2% chickpeas).

To differentiate the coffee samples, principal component analysis (PCA) was applied. The model extracted two components with eigenvalues greater than 1, which explained 83.6% of the total variance (Supplementary Materials Table S11). The first principal component was loaded with Pr, Nd, Sm, Gd, Tb, Dy, Ho, Er, Y, Ce, and La, accounting for 68.9% of the total variance. The second principal component explained 14.7% of the total variance and had a strong loading of Eu, Tm, Yb, Lu, and Sc. The score plots for the first two components are presented in Figure 4. The score plot shows the separation of coffees with additives on one side (orange and violet samples) and roasted ground coffees and roasted ground coffees in capsules on the other side (pink and blue samples). Two samples of coffees with 2% chickpeas (red star) were closest to the roasted ground coffee samples. On the other hand, the surrogates for coffee (red dots) were separated from all other coffee samples. The PCA results are important in distinguishing coffee samples from surrogate samples and identifying coffee with added surrogates. This analysis not only underscores the variations in composition but also aids in identifying potential adulteration in coffee products.

**Figure 5:**
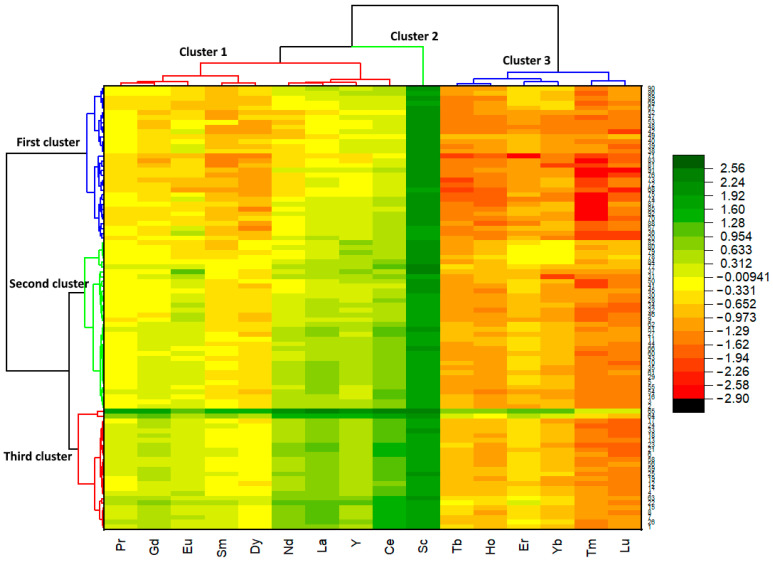
Heatmap dendrogram for the coffee samples and rare earth elements (REEs).

Hierarchical cluster analysis (HCA) revealed three clusters of coffee samples (Figure 5). The first cluster (blue) comprised 34.8% of the analyzed samples. This cluster mostly represented instant coffees and instant coffees with additives, which were separated into two sub-clusters. The second (green) and third clusters (red) comprised 38.0% and 27.2% of the analyzed samples, respectively. The second cluster mostly included ground roasted coffee and ground roasted coffee in capsules but also included several instant coffees and instant coffees with additives.

Instant coffees with additives from the second cluster generally contained chocolate as an ingredient. Additionally, 28.6% of the coffee in the second cluster was labeled with 100% Arabica, while 20% had a geographic origin. The last data are likely consistent with the fact that these samples generally have lower REE content, which is why they are positioned in the same cluster. The third cluster consisted of ground roasted coffee and two samples of ground roasted coffee with surrogates, one with 2% chickpeas and the other with 10% barley. Most samples in this cluster represented a blend of two coffee species, Arabica and Robusta, even in the case of coffees containing substitutes. This cluster also had a right sub-cluster that included only two samples that were coffee substitutes (100% chicory and 70% barley + 30% chicory).

The HCA identified three REE clusters. Cluster 1: Pr, Gd, Eu, Sm, Dy; Cluster 2: Nd, La, Y, Ce, Sc; Cluster 3: Tb, Ho, Er, Yb, Tm, Lu. Most samples showed concentrations in the order of Cluster 2 > Cluster 1 > Cluster 3, meaning Ce, La, Sc, Nd, and Y were most abundant, followed by Pr, Gd, Eu, Sm, and Dy, with Tb, Ho, Er, Yb, Tm, and Lu being the least abundant.

**Figure 6:**
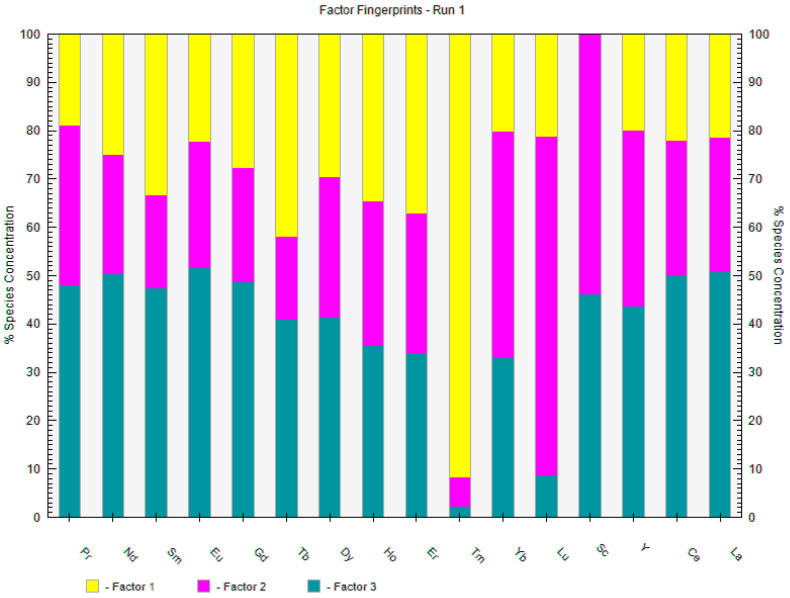
PMF results illustrate the contribution of REEs to three different factors.

The positive matrix factorization method (PMF) is often used in environmental studies to characterize natural and anthropogenic pollution sources in soil [54], sediment [55], and water [18]. Alongside correlation analysis, PCA, HCA, and PMF can help identify different behaviors and potential sources of REEs in coffee samples. In our study, the PMF model was run 20 times, with the number of factors set between 3 and 5. The best results were obtained for three factors (Figure 6), yielding the smallest Q value. However, the coefficients of the observed and predicted values (R^2^) varied. Most REEs had R^2^ values above 0.81, except for Yb, Eu, and Sc, which had R^2^ values of 0.69, 0.52, and 0.041, respectively.

Factor 1 was characterized by Tm, Tb, and Er; Factor 2 by Lu, Sc, and Yb; and Factor 3 was associated with the remaining elements (Pr, Nd, Sm, Eu, Gd, Dy, Ho, Y, Ce, and La). Our classification of REEs based on the identified factors is quite similar to the classification of REEs using PCA in a study by de Oliveira Costa et al. (2024) [35]. The formation of a distinct group comprising Lu, Tm, and Yb was observed. Elements that stand out are the most significant for sample separation. Additionally, in Vezzulli et al.’s study (2023) [30], which explored the elemental profiling and origin of specialty and high-quality coffees, Tm was identified as the significant discriminant element among others. Previous research has noted that Lu and Yb exhibit the same trend in plants as in terrestrial matrices, meaning that plants reflect the soil composition [56]. Artificial fertilizers lead to an increase in REEs in soil and, consequently, in coffee. Special coffee varieties are generally expected to have lower REE concentrations due to careful cultivation and better control of artificial fertilizer addition [35]. Furthermore, an increase in REEs in final coffee samples can occur due to the addition of surrogates. A study from 2019 that investigated the REE content in tap water and beverages from fast-food franchises revealed that Coca-Cola beverages are characterized by a strong enrichment of Yb and Lu derived from syrup [57].

Based on our analysis, it is not possible to unambiguously determine the origin of the coffee samples, as they consist of various coffee types, blends, and samples with different additives. However, clear distinctions were observed in cases involving surrogate additives (e.g., barley, chickpea) and between most of the different coffee types (ground versus instant). Our findings also suggest the potential for classifying coffee samples based on their origins or varieties (e.g., Arabica versus Robusta). This highlights that the analysis of rare earth elements (REEs), combined with multivariate analysis techniques, could be valuable for authenticating unmodified or minimally processed coffee samples, providing a foundation for future research. Furthermore, the assessment of REE content could serve as a tool for detecting potential adulteration in coffee products.

### 3.4. Potential REE Hazards to Human Health

**Figure 7:**
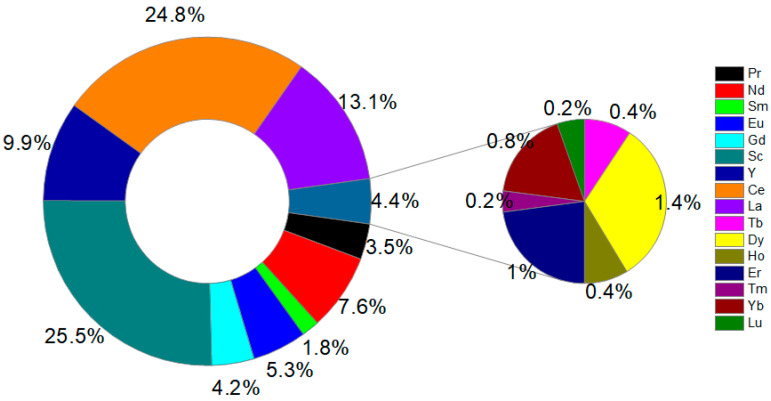
Contribution of REEs to the hazard index (HI) and target cancer risk (TCR) from coffee.

The potential health effects of REEs in coffee were assessed using the model previously applied for heavy metal(loid)s. This model incorporates ingestion exposure pathways. The calculated hazard quotient (HQ) values follow a decreasing order (mean) as follows: Ce (6.77 × 10^−8^) > La (3.43 × 10^−8^) > Sc (2.70 × 10^−8^) > Y (2.55 × 10^−8^) > Nd (2.37 × 10^−8^) > Gd (1.25 × 10^−8^) > Eu (8.03 × 10^−9^) > Pr (7.67 × 10^−9^) > Sm (5.26 × 10^−9^) > Dy (4.26 × 10^−9^) > Er (2.52 × 10^−9^) > Yb (1.65 × 10^−9^) > Tb (1.10 × 10^−9^) > Ho (9.25 × 10^−10^) > Lu (3.78 × 10^−10^) > Tm (3.64 × 10^−10^). The min–max HQ values for REEs in coffee are provided in Table 4. Based on the resulting HQ, HI, and TCR values, one may assume that REEs pose a negligible health risk to coffee consumers. A previous study by Savić et al. (2024) [7] evidenced that the adverse health effects of coffee are predominantly driven by the presence of HMs rather than REEs. The contribution of REEs to HI and TCR is presented in Figure 7. Scandium and Ce were responsible for 25.5% and 24.8% of non-carcinogenic and carcinogenic risks.

## 4. Conclusions

This study describes the levels of 14 rare earth elements (REEs) (La, Ce, Pr, Nd, Sm, Eu, Gd, Tb, Dy, Ho, Er, Tm, Yb, Lu), and Y and Sc in 92 coffee samples. The ultrasound-assisted extraction (UAE) procedure was developed for the extraction of REEs; subsequently, their determination was performed by ICP-MS. This study confirmed that the UAE procedure is an efficient and accurate method for preparing coffee samples for further determination of REEs. The most abundant REE was Ce, followed by La, Sc, Y, Nd, Gd, Eu, Pr, Sm, Dy, Er, Yb, Tb, Ho, Lu, and Tm. Among the analyzed elements, Tb was below the LOD value in 4 samples; Sm, Ho, and Er were below the LOD values in 5 samples; while Dy, Tm, and Lu were below the corresponding LOD values in 8, 9, and 10 samples, respectively. The surrogates had significantly higher total REE content than the pure coffee samples. The multivariate analysis techniques effectively distinguished between different types of coffee, clearly separating surrogates from 100% coffee samples. Principal component analysis (PCA) highlighted Eu, Tm, Yb, Lu, and Sc as significant elements of separation, which was similarly confirmed by the positive matrix factorization method (PMF), where there was a distinct separation of Tm, Tb, and Er, as well as Lu, Sc, and Yb from other elements. PMF analysis suggested that coffee reflects the soil composition. An increase in REEs in coffee can result from the addition of surrogates and the use of artificial fertilizers.

In conclusion, future research should investigate the potential for authenticating raw and minimally processed coffee based on the content of REEs. Furthermore, additional studies are needed to explore the applicability of this method to other types of food matrices and to evaluate the long-term health risks associated with REE exposure.

## Figures and Tables

**Table 1:** Instrument operating conditions for ICP-MS.

Parameter	Value
Forward power	1550 W
Nebulizer gas (Ar)	0.99 L/min
Auxiliary gas (Ar)	0.8 L/min
Cool gas flow	14 L/min
Collision cell gas (He)	4.5 mL/min
Sample uptake/wash time	45 s each
Dwell time	0.01 s
Repeats per sample	3
Pump speed	40 rpm

**Table 2:** Calibration parameters for ICP-MS of REEs.

*m*/*z*	Element	Polyatomic Interferences	LOD, ng/L	R^2^	Linear Equation
141	Pr	/	0.19	0.9962	f(x) = 48.6 × 10^3^x + 1.67
146	Nd	BaO, RuO_3_	1.38	0.9970	f(x) = 13.6 × 10^3^x + 1.33
147	Sm	RuO_3_	0.52	0.9999	f(x) = 68.8 × 10^3^x + 1.45
153	Eu	BaO	0.19	0.9986	f(x) = 33.8 × 10^3^x + 2.19
157	Gd	CeO, PrO, LaO, BF	0.50	0.9989	f(x) = 69.1 × 10^3^x + 0.70
159	Tb	NdO, PrO	0.14	0.9981	f(x) = 45.9 × 10^3^x+ 3.27
163	Dy	NdO, SmO	0.62	0.9998	f(x) = 11.3 × 10^3^x + 0.33
165	Ho	SmO	0.16	0.9964	f(x) = 44.0 × 10^3^x + 1.45
166	Er	SmO, NdO	0.48	0.9955	f(x) = 14.9 × 10^3^x + 1.01
169	Tm	EuO	0.04	0.9977	f(x) = 41.0 × 10^3^x + 0.45
172	Yb	DyO, SmO, GdO	0.02	0.9997	f(x) = 19.6 × 10^3^x + 0.79
175	Lu	GdO, TbO	0.12	0.9982	f(x) = 40.7 × 10^3^x + 3.40
45	Sc	CO_2_, SiO, BO_2_, AlO,	4.09	0.9982	f(x) = 16.4 × 10^3^x + 1.02
89	Y	CaH, CHO_2_, SiOH, N_2_OH	0.91	0.9985	f(x) = 38.5 × 10^3^x + 4.70
140	Ce	/	0.44	0.9987	f(x) = 38.0 × 10^3^x + 20.7
139	La	/	0.30	0.9988	f(x) = 44.1 × 10^3^x + 37.3

f(x): instrument response in cps (counts per second); x: analyte concentration in µg/L.

**Table 3:** The range of REE concentrations (µg/kg) in the coffee samples from other studies compared to the present study.

REEs	Vezzulli et al. (2023) [30]	de Oliveira Costa et al. (2024) [35] ^1^	Barbosa et al. (2014) [29]	Messaoudi et al. (2018) [53]	Santato et al. (2012) [3]	Present Study ^$^	Present Study ^#^
Pr	<2.12–2.74 ^2^; 8.96 ^3^; 2.71 ^5^; 13.61 ^6^	3–200	-	-	0.16–3.38	0.27–3.58	0.89–39.7
Nd	<3.12–11.9 ^1^; 38.2 ^3^; 3.5 ^4^; 13.2 ^5^; 76.8 ^6^; 3.9 ^7^	10–700	-	-	0.38–12.1	<LOD–15.9	2.13–148
Sm	<3.99–9.26 ^3^; 8.8 ^6^	3–400	3.5–62	7.8–8.3 ^a^; 4.0–4.3 ^b^	0.20–2.36	<LOD–3.46	0.62–33.8
Eu	<3.93–4.3 ^6^	1–10	1.3–15.1	nd ^a^; nd–11.7 ^b^	0.13–1.02	0.090–12.9	0.83–10.1
Gd	<3.71–7.57 ^3^; 6.9 ^6^	3–60	0.9–24.9	-	0.18–2.39	0.092–7.59	1.18–76.2
Tb	<3.25	1–10	0.07–10.6	-	-	<LOD–0.57	0.096–6.68
Dy	<3.94–7.4 ^3^; 9.9 ^6^	2–30	0.4–39.3		0.17–1.84	<LOD–1.98	0.42–31.3
Ho	<2.79	1–10	0.11–6.5	-	-	<LOD–0.38	0.072–5.69
Er	<2.66–4.0 ^3^; 3.4 ^6^	2–20	0.3–11.1	-	0.21–1.07	<LOD–1.00	0.25–16.1
Tm	<2.23	1–8	-	-	<LOD–0.21	<LOD–0.15	0.043–1.83
Yb	<3.82	2–10	0.1–9.2	-	<LOD–0.94	0.010–0.89	0.17–9.74
Lu	<2.46	3–20	0.13–0.72	-	-	<LOD–0.26	0.040–1.42
Ce	<2.55–23.2 ^1^; 4.0 ^2^; 64.2 ^3^; 6.8 ^4^; 28.5 ^5^; 114.5 ^6^; 9.4 ^7^	10–2100	9.5–361	74–89 ^a^; 490–510 ^b^	0.73–36.2	0.81–32.9	7.58–347
La	<2.40–23.1 ^1^; 4.9 ^2^; 34.8 ^3^; 5.3 ^4^; 15.2 ^5^; 85.9 ^6^; 7.1 ^7^	10–1000	3.2–122	35–36 ^a^; 85–91 ^b^	0.59–15.5	0.37–17.1	3.45–186
Sc	-	10–40	-	37–38 ^a^; 66–68 ^b^	-	2.09–10.6	3.81–14.5
Y	-	3–100	-	-	-	0.31–10.4	2.33–169

^1^—Brazil; ^2^—Columbia; ^3^—Panama; ^4^—Costa Rica; ^5^—Ethiopia; ^6^—India; ^7^—Indonesia; ^a^—Arabica; ^b^—Robusta. ^$^—coffee samples; ^#^—surrogates and coffee with the addition of surrogates (2% chickpeas or 10% barley).

**Table 4:** Hazard quotients (HQs), hazard index (HI), and target cancer risk (TCR) for REEs in coffee.

Health Risk Index	REE	Mean	Min	Max
HQ	Ce	6.77 × 10^−8^	4.69 × 10^−9^	2.00 × 10^−6^
	La	3.43 × 10^−8^	2.11 × 10^−9^	1.07 × 10^−6^
	Sc	2.70 × 10^−8^	1.20 × 10^−8^	8.38 × 10^−8^
	Y	2.55 × 10^−8^	1.80 × 10^−9^	9.74 × 10^−7^
	Nd	2.37 × 10^−8^	9.29 × 10^−10^	8.50 × 10^−7^
	Gd	1.25 × 10^−8^	5.31 × 10^−10^	4.39 × 10^−7^
	Eu	8.03 × 10^−9^	5.17 × 10^−10^	7.42 × 10^−8^
	Pr	7.67 × 10^−9^	1.56 × 10^−9^	2.29 × 10^−7^
	Sm	5.26 × 10^−9^	1.45 × 10^−10^	1.95 × 10^−7^
	Dy	4.26 × 10^−9^	1.82 × 10^−10^	1.80 × 10^−7^
	Er	2.52 × 10^−9^	1.42 × 10^−11^	9.30 × 10^−8^
	Yb	1.65 × 10^−9^	5.90 × 10^−11^	5.61 × 10^−8^
	Tb	1.10 × 10^−9^	4.52 × 10^−11^	3.85 × 10^−8^
	Ho	9.25 × 10^−10^	6.79 × 10^−11^	3.27 × 10^−8^
	Lu	3.78 × 10^−10^	7.30 × 10^−11^	8.20 × 10^−9^
	Tm	3.64 × 10^−10^	1.09 × 10^−11^	1.06 × 10^−8^
HI	ΣREEs	2.23 × 10^−7^	4.70 × 10^−8^	6.32 × 10^−6^
TCR	ΣREEs	1.24 × 10^−13^	2.61 × 10^−14^	3.51 × 10^−12^

## Data Availability

The original contributions presented in the study are included in the article/Supplementary Materials, further inquiries can be directed to the corresponding author.

## Supplementary Materials

The following supporting information can be downloaded at https://www.mdpi.com/article/10.3390/foods14020275/s1: Table S1: Coffee samples analyzed in this study; Table S2: Equations used in the risk assessment model; Table S3: Exposure parameters and their distributions in the risk assessment model; Table S4: Concentration of REEs in coffee samples (*n* = 10) when prepared by MAD and UAE procedures (µg/kg); Table S5: Correlation coefficients (R^2^) of the linear regression (MAD vs. UAE); Table S6: Analysis of certified reference material (SRM 1547) for the content of REEs (mean ± SD, n = 3); Table S7: Initial concentrations (µg/kg) of REEs in spiked samples; Table S8a: Recovery and precision for spiking of UAE experiments (Spike 1: 0.5 µg/kg; Spike 2: 5.0 µg/kg); Table S8b: Recovery and precision for spiking of UAE experiments (Spike 3: 40 µg/kg; Spike 4: 200 µg/kg); Table S8c: Recovery and precision for spiking of MAD experiments (Spike 1: 0.5 µg/kg; Spike 2: 5.0 µg/kg); Table S8d: Recovery and precision for spiking of MAD experiments (Spike 3: 40 µg/kg; Spike 4: 200 µg/kg); Table S9: Descriptive statistics of the REE concentrations in coffee; Table S10: Pearson correlation matrix for REEs in coffee samples; Table S11: Principal component analysis showing two extracted components with eigenvalues greater than one, and their loadings.
