# Supplementary material for: Ultrasound-Assisted Extraction Followed by Inductively Coupled Plasma Mass Spectrometry and Multivariate Profiling of Rare Earth Elements in Coffee
Source: Foods. 2025 Jan 16;14(2):275. doi: 10.3390/foods14020275 (PMC11764531; doi:10.3390/foods14020275)
Supplement: Supplementary file 1 [file foods-14-00275-s001.zip › foods-3388262-supplementary.pdf]

Supplementary material

Ultrasound-assisted extraction followed by ICP-MS and multivariate profiling of rare earth elements in coffee

Aleksandra Savić[1], Jelena Mutić[2], Milica Lučić[3], Jelena Vesković[4], Andrijana Miletić[4], and Antonije Onjia[4,*]

[1] Anahem Laboratory, Mocartova 10, 11160 Belgrade, Serbia;
[2] Department of Analytical Chemistry, Faculty of Chemistry, University of Belgrade, 11158 Belgrade, Serbia;
[3] Innovation Center of the Faculty of Technology and Metallurgy, 11120 Belgrade, Serbia;
[4] Faculty of Technology and Metallurgy, University of Belgrade, 11120 Belgrade, Serbia;
***** Correspondence: onjia@tmf.bg.ac.rs

Tabela S1. Coffee samples analyzed in this study.

No	Producer	Brand	Name of coffee	Description	Coffee type	Country origin	Country producer
1	Strauss Adriatic	Doncafe	Doncafe Moment	Extra fine ground traditional black coffee	Arabica, Robusta	****	Serbia
2	Strauss Adriatic	Doncafe	Doncafe Strong Moja jutarnja snaga	A blend of roasted ground coffee	Arabica, Robusta	****	Serbia
3	Strauss Adriatic	Doncafe	Doncafe Minas	A blend of roasted ground coffee	100% Arabica	****	Serbia
4	Strauss Adriatic	Doncafe	Premium Coffe Single origin, Uganda	Roasted ground coffee	100% Arabica	Uganda	Serbia
5	Strauss Adriatic	Doncafe	Premium Coffe Single origin, Brazil	Roasted ground coffee	Arabica, Robusta	Brazil	Serbia
6	Atlantic Grand	Grand	Gold	A blend of roasted ground coffee	Arabica, Robusta	****	Serbia
7	Atlantic Grand	Grand	Grand Strong	A blend of roasted ground coffee	Arabica, Robusta	****	Serbia
8	Atlantic Grand	Grand	Aroma	A blend of roasted ground coffee	100% Arabica	****	Serbia
9	Atlantic Droga Kolinska - Grand	Grand	Single Origin, Rwanda Bourbon	Roasted ground coffee	100% Arabica	Ruanda	Slovenia
10	Atlantic Droga Kolinska - Grand	Grand	Single Origin, Brazil Cerrado	Roasted ground coffee	Arabica, Robusta	Brazil	Slovenia
11	Strauss Adriatic	C kafa	Zlatna	A blend of roasted ground coffee	Arabica, Robusta	****	Serbia
12	Strauss Adriatic	C kafa	C kafa	A blend of roasted ground coffee	Arabica, Robusta	****	Serbia
13	Kafa Dobro Jutro	Dobro jutro	Dobro Jutro	A blend of roasted ground coffee	Arabica, Robusta and 10% barley	****	Serbia
14	Kafa Dobro Jutro	Dobro Jutro	Minas	A blend of roasted ground coffee	Arabica, Robusta	****	Serbia
15	Atlantic Grand	Bonito	Bonito prava kafa	A blend of roasted ground coffee	Arabica, Robusta	Brazil	Serbia
16	Moravka Pro	Moravka	CafeKafica Gold	A blend of roasted ground coffee	Arabica, Robusta	****	Serbia
17	Jacobs Douwe Egberts	Jacobs	Monarch	A blend of roasted ground coffee	Arabica, Robusta	****	Greece
18	****	Zlatno Zrno	Turska kafa, Strong	A blend of roasted ground coffee	100% Arabica	****	Europe
19	SZTR ALLCAFE	Allcafe	Kraljica Premium	A blend of roasted ground coffee	85% Arabica, 15% Robusta	Brazil, Columbia, Guatemala, India	Serbia
20	For Lidl Srbija	SUNGA	Kafa, mlevena	A blend of roasted ground coffee	Arabica, Robusta	Brazil	*
21	Franch	Franch	Jubilarna original	A blend of roasted ground coffee	Arabica, Robusta	Middle and South America, Asia	Croatia
22	Strauss Adriatic	Doncafe	Doncafe 3sec, 2,1 Black	A blend of roasted ground coffee	Arabica, Robusta	****	Serbia
23	Atlantic Grand	Grand	Grand Black n easy	A blend of roasted ground coffee and coffee extract	****	****	Serbia
24	Atlantic Grand	Grand	Black n easy Strong	A blend of roasted ground coffee	Arabica, Robusta	****	Serbia
25	Kafa Dobro Jutro	Dobro jutro	U maloj kesici - Dobro Jutro Prava domaća	A blend of roasted ground coffee	Arabica, Robusta	****	Serbia

26	For Lidl Srbija	Bellarom	ROSSO	A blend of roasted ground coffee	100% Arabica	****	Germany
27	Utramar Caffe Srl za Atlantic Droga Kolinska	BarCaffe	Perfetto Single Origin Brazil	Roasted ground coffee in capsules	****	Brazil	Italy
28	Jacobs Douwe Egberts	Jacobs	Espresso 7 Classico	A blend of roasted ground coffee in capsules	Arabica, Robusta	****	Netherlands
29	Utramar Caffe Srl za Atlantic Droga Kolinska	BarCaffe	Espresso	A blend of roasted ground coffee in capsules	100% Arabica	****	Italy
30	BarCaffe/Ultamar Caffe Srl	BarCaffe	Perfetto Single Origin Ethiopia	Roasted ground coffee in capsules	****	Ethiopia	Italy
31	Jacobs Douwe Egberts	Jacobs	Espresso 10 Intenso	A blend of roasted ground coffee in capsules	100% Arabica	****	Netherlands
32	For Lidl Srbija	Bellarom	Classico espresso	Roasted ground coffee in capsules	Arabica	****	****
33	For Lidl Srbija	Bellarom	Kafa mlevena	Roasted ground coffee	100% Arabica	****	****
34	For Lidl Srbija	Bellarom	Forte Lungo	Roasted ground coffee in capsules	100% Arabica	****	Germany
35	For Lidl Stiftung, Germany	Bellarom	Gold Lungo	Roasted ground coffee in capsules	100% Arabica	****	****
36	For Lidl Stiftung, Germany	Bellarom	Ristretto espresso	Roasted ground coffee in capsules	100% Arabica	****	****
37	For Lidl Srbija	Bellarom	Barista Origins Tanzania	Instant coffee, lyophilized	100% Robusta	Tanzania	Vietnam
38	For Lidl Srbija	Bellarom	Exclusive	Instant coffee, lyophilized	80% Robusta, 20% Arabica	****	Croatia
39	For Lidl Stiftung, Germany	Bellarom	Gold Decaf	Instant coffee, lyophilized	100% Robusta	****	****
40	For Lidl Srbija	Bellarom	Barista Origins Sumatra	Instant coffee, lyophilized	60 % Robusta, 40 % Arabica	Indonesia	India
41	For Lidl Bulgaria	Bellarom	Mild, mild aroma	Instant coffee, agglomerated	80% Robusta, 20% Arabica	****	****
42	Bellarom/Lidl Srbija	Bellarom	Gold Classic	Instant coffee, lyophilized	80% Robusta, 20% Arabica	****	Bulgaria
43	For Lidl Srbija	Lidl srbija	Pržena mlevena kafa	A blend of roasted ground coffee	****	****	Slovenia
44	Costa Coffee	Costa coffee	Smooth and nutty, Signature Blend, Lungo	A blend of roasted ground coffee in capsules	****	****	Italy
45	For Aman doo.	My cup	Instant coffee	Instant coffee, agglomerated	****	India	India
46	Nestle UK LTD, England	Starbucks blonde	Espresso roast	A blend of roasted ground coffee in capsules	****	South America	England
47	Nestle France za Nestle Adriatic doo	Nescafe	Nescafe Crema	Instant coffee	****	****	France
48	Nestle Espana S.A.	Nescafe	Nescafe Decaf	Instant coffee without caffeine	****	****	Spain
49	Espana S.A./ Nestle Hungaria/Nestle Adriatic	Nescafe	Nescafe Classic	Instant coffee	****	****	Spain
50	****	Jacobs	Crema	Instant coffee	****	Columbia, Vietnam	Germany
51	Nestle Germany	Nescafe	Dolce Gusto, espresso inenso 7	A blend of roasted ground coffee in capsules	****	****	Germany
52	Nestle Espana S.A. For Nestle Adriatic	Nescafe	Black Roast Intense Taste	Instant coffee	****	****	****
53	SEDA Outspan Iberia for Atlantic Grand	Grand	Insta Grand, Classic	Instant coffee granules	100% Arabica	****	Spain
54	Atlantic Droga Kolinska - Grand	Grand	Grand Brazil Cerrado single origion	Roasted ground coffee	100% Arabica	Brazil	Slovenia
55	Kalča	Kalča	Kalča kafa	A blend of roasted ground coffee	60% Arabica, 40% Robusta	****	Serbia
56	Mercator S/Moravka Pro	Mercator S	Ritual coffee selection	A blend of roasted ground coffee	Arabica, Robusta	****	****
57	Atlantic Grand	Grand	Peru Chanchamayo single origin	Roasted ground coffee	100% Arabica	Peru	Serbia
58	Atlantic Grand	Grand	100% Arabica Gourment kafa	Roasted ground coffee	100% Arabica	****	Serbia
59	Atlantic Grand	Bonito	Bonito prava kafa, tamno pržena	Roasted ground coffee	****	Brazil	Serbia
60	Gradska pržionica, Gučevska 10, Beograd	Pržioničar	Pržioničar Jaka	A blend of roasted ground coffee	Arabica, Robusta	****	Serbia

61	Gradska pržionica, Gučevska 10, Beograd	Pržioničar	Pržioničar Klasik	A blend of roasted ground coffee	Arabica, Robusta	****	Serbia
62	Strauss Adriatic	Doncafe	Green active	A blend of roasted ground coffee with extract of green coffee	97% blend of ground roasted coffee + 3% coffee extract	****	Serbia
63	Amigos kafa	Amigos	Domaća mlevena kafa Speciale	Coffee-based product with chickpeas	Minas coffee with 2% chickpeas	Brazil	Serbia
64	Franck	Franck	Divka original	Fried barley and chicory root	70% barley and 30% chicory	Croatia	Croatia
65	Franck	Franck	Cikorija Kadovina	Fried chicory root	100% chicory	Croatia	Croatia
66	Amigos kafa	Amigos	Proizvod na bazi kafe sa slanutkom	Ground fried product with chickpeas based on coffee	80% Arabica, 18% Robusta, 2% chickpeas	****	Serbia
67	Nestle Hungaria	Nescafe	Nescafe, 3in1 Brown sugar (Smuth & rich)	Instant powder for preparing an instant coffee drink with brown sugar	Instant coffee 8%	****	Hungary
68	Nestle Hungaria	Nescafe	Nescafe, 3in1, Black roast	Instant powder for preparing an instant coffee drink	Instant coffee 14.6%	****	Hungary
69	Nestle Hungaria	Nescafe	Nescafe, 3in1, Strong (Smuth & rich)	Instant powder for preparing an instant coffee drink	Instant coffee 15.7%	****	Hungary
70	Nestle Hungaria	Nescafe	Nescafe, 3in1, Creamy latte (Smuth & rich)	Instant powder for preparing an instant coffee drink	Instant coffee 4.6%	****	Hungary
71	Nestle	Nescafe	Nescafe, 3in1, Frappe Caramel	Instant powder for preparing instant coffee drink with caramel flavor	Instant coffee 8%	****	****
72	Nestle	Nescafe	Milky and foamy, Nescafe Latte	A powdered mixture of coffee extract with additives, with an intense milky taste	Instant coffee 8.9%	****	****
73	Nestle	Nescafe	Reach and foamy, Nescafe cappuccino	A powdered mixture of coffee extract with additives, with an intense milky taste	Instant coffee 6.6%	****	****
74	Nestle Suisse	Nescafe	Nescafe Gold	Instant coffee	****	****	Switzerland
75	Nestle Espana	Nescafe	Nescafe Classic	Instant coffee	****	****	Spain
76	Atlantic Grand	Grand	Insta Grand Cappuccino Vanilla	Powdered blend of coffee extract with vanilla flavor	Instant coffee 9%	****	Serbia
77	Atlantic Grand	Grand	Insta Grand Cappuccino Choco	Powdered mixture of coffee extract with chocolate aroma	Instant coffee 4.5%	****	Serbia
78	Atlantic Grand	Grand	Insta Grand 3n1 Choco orange	Powdered mixture of coffee extract with chocolate and orange flavor	Instant coffee 9%	****	Serbia
79	Atlantic Grand	Grand	Insta Grand 2n1 Slim and fit biotin	Powdered mixture of coffee extract with coenzyme Q10, L-kamitin and biotin	Instant coffee 7%	****	Serbia
80	Atlantic Grand	Grand	Insta Grand 3n1 Choco Banana	Powdered mixture of coffee extract with chocolate and banana flavor	Instant coffee 5%	****	Serbia
81	Atlantic Grand	Grand	Insta Grand Capuccino Classic	Powdered blend of coffee extract with additives	Instant coffee 10%	****	Serbia
82	Atlantic Grand	Grand	Insta Grand 2in1 Classic	Powdered blend of coffee extract with Irish cream flavor	Instant coffee 19%	****	Serbia
83	Atlantic Grand	Grand	Insta Grand Cappuccino Irish Cream	Powdered mixture of coffee extract with chocolate aroma	Instant coffee 6%	****	Serbia
84	Atlantic Grand	Grand	Insta Grand 3in1 Najlepše želje	Powdered blend of coffee extract with additives	Instant coffee 5%	****	Serbia
85	Atlantic Grand	Grand	Insta Grand Freeze Classic	Instant coffee drink mix	Instant coffee 6%	****	Serbia
86	Jacobs Douwe Egberts	Jacobs	Jacobs Original 3in1	A mixture of instant coffee with additives	Instant coffee 15%	****	Netherlands
87	Jacobs Douwe Egberts	Jacobs	Iced Cappuccino Salted Caramel	A mixture of instant coffee with additives	Instant coffee 5.6%	****	The Czech Republic
88	Jacobs Douwe Egberts	Jacobs	Iced Cappuccino Original	Instant coffee drink mix	Instant coffee 5.6%	****	The Czech Republic
89	Jacobs Douwe Egberts	Jacobs	Jacobs Original 2in1	Powdered blend of coffee extract with additives	Instant coffee 9.9%	****	the Czech Republic
90	Moravka Pro	Moravka	cafeKafica Instant mix 2in1	Powdered blend of coffee extract with additives	Instant coffee 19%	****	Serbia
91	Fruitica				Instant coffee 7%	****	Serbia
92	Fruitica	K plus	3in1 coffee mix	Powdered blend of coffee extract with additives	Instant coffee 18%	****	Serbia

**** data not available

Table S2. Equations used in the risk assessment model.

Average Daily Dose
$$ADD = \frac{C \times IngR \times EF \times ED}{BW \times AT} \times CF$$
Non-Carcinogenic Risk
$$HQ = \frac{ADD}{RfD}$$ $$HI = \sum_{i=1}^{n} HQ_i$$
Carcinogenic Risk
$$CR = ADD \times CSF$$ $$TCR = \sum_{i=1}^{n} CR_i$$
The risk assessment model parameters: C (element concentration), IngR (ingestion rate), EF (exposure frequency), ED (exposure duration), BW (body weight), ATnc (average non-carcinogenic time), ATc (average carcinogenic time), CF (conversion factor), RfD (reference dose), CSF (cancer slope factor).

Table S3. Exposure parameters and their distributions in the risk assessment model.

Exposure parameter	Abbrev.	Unit	Adults
Element concentration	C	$mg \cdot kg^{-1}$	-
Ingestion rate	IngR	$mg \cdot day^{-1}$	-
Exposure frequency	EF	$day(s) \cdot year^{-1}$	350
Exposure duration	ED	year(s)	24
Body weight	BW	kg	70
Average non-carcinogenic time	ATnc	day(s)	8760
Average carcinogenic time	ATc	day(s)	25,550
Conversion factor	CF	$kg \cdot mg^{-1}$	10^{-6}
Reference dose	RfD	$mg \cdot kg^{-1} bw \cdot day^{-1}$	-
Cancer slope factor	CSF	$kg \cdot bw \cdot day \cdot mg^{-1}$	-

Table S4. Concentration of REEs in coffee samples (n=10) when prepared by MAD and UAE procedures (µg/kg).

UAE	Pr	Nd	Sm	Eu	Gd	Tb	Dy	Ho	Er	Tm	Yb	Lu	Sc	Y	Ce	La
1	1.64	6.95	1.30	1.52	3.34	0.227	0.680	0.127	0.450	0.048	0.221	0.052	3.37	3.90	21.2	8.87
2	0.610	2.52	0.600	1.31	1.18	0.130	0.276	0.073	0.101	0.036	0.130	0.036	2.69	2.08	8.61	4.22
3	0.548	2.10	0.502	1.26	0.910	0.112	0.298	0.060	0.143	0.032	0.134	0.033	2.46	2.18	6.25	3.74
4	1.08	4.34	0.964	2.73	1.80	0.159	0.566	0.146	0.281	0.054	0.161	0.039	2.49	3.86	12.9	5.97
5	0.802	3.02	1.09	2.04	1.18	0.106	0.386	0.092	0.357	0.034	0.158	0.043	2.29	3.05	8.21	5.39
6	1.50	6.22	1.05	1.78	3.27	0.169	0.499	0.094	0.322	0.056	0.175	0.021	2.52	3.29	20.2	8.24
7	1.93	7.43	1.59	2.15	3.82	0.220	0.504	0.184	0.275	0.054	0.195	0.051	2.66	3.94	24.3	10.1
8	1.81	7.89	1.25	1.92	3.52	0.277	0.849	0.095	0.288	0.055	0.141	0.047	2.48	3.88	24.8	9.98
9	0.331	1.06	1.59	2.50	1.64	0.075	0.134	0.061	0.095	0.022	0.064	0.049	2.09	1.36	2.44	1.46
10	0.773	2.99	0.569	1.13	1.12	0.107	0.344	0.052	0.338	0.030	0.125	0.023	2.29	2.22	7.68	5.13
MAD	Pr	Nd	Sm	Eu	Gd	Tb	Dy	Ho	Er	Tm	Yb	Lu	Sc	Y	Ce	La
1	2.49	6.72	1.27	1.60	3.00	0.162	0.590	0.090	0.505	0.041	0.266	0.053	5.33	3.73	26.1	12.4
2	0.934	3.09	0.401	1.38	1.19	0.126	0.170	0.065	0.152	0.040	0.191	0.055	4.21	2.21	10.8	4.31
3	0.808	1.92	0.357	1.24	0.875	0.103	0.292	0.051	0.214	0.038	0.187	0.058	4.07	2.06	7.27	3.59
4	1.68	5.18	1.13	2.30	2.52	0.176	0.530	0.186	0.432	0.036	0.253	0.058	4.37	3.84	17.3	7.95
5	1.01	2.53	0.690	1.95	1.13	0.090	0.340	0.091	0.329	0.025	0.173	0.061	3.57	3.03	9.56	5.76
6	2.13	6.03	1.01	1.45	2.66	0.175	0.455	0.083	0.438	0.039	0.173	0.038	4.08	3.56	24.9	10.1
7	2.46	6.89	1.30	1.98	3.31	0.264	0.578	0.150	0.289	0.036	0.227	0.041	4.38	3.76	29.1	12.1
8	2.83	7.70	1.82	1.65	3.17	0.210	0.687	0.126	0.434	0.060	0.178	0.045	4.13	4.57	31.5	13.9
9	0.501	1.57	0.232	2.58	0.456	0.051	0.194	0.097	0.139	0.022	0.091	0.052	3.17	1.67	4.04	2.10
10	1.13	3.32	0.425	1.14	1.43	0.106	0.284	0.072	0.203	0.044	0.136	0.042	3.82	2.58	8.98	5.98

Table S5. Correlation coefficients (R^2) of the linear regression (MAD vs. UAE).

	R^2
Pr	0.9664
Nd	0.9969
Sm	0.2081
Eu	0.8866
Gd	0.7974
Tb	0.8459
Dy	0.8200
Ho	0.5967
Er	0.6909
Tm	0.3470
Yb	0.8008
Lu	0.1679
Sc	0.9034
Y	0.9344
Ce	0.9831
La	0.9481

Table S6. Analysis of certified reference material (SRM 1547) for the content of REEs (mean ± SD, n=3).

Element	Certified value (µg/kg)	Obtained concentration (µg/kg)	Margin of error (µg/kg)	95% CI Lower (µg/kg)	95% CI Upper (µg/kg)	RSD (%)	Recovery (%)
Ce	10,000	9575±350	868	8705	10444	3.65	95.7
Eu	170	175±17	42.2	133	217	9.71	103
Gd	1000	1110±124	308	802	1418	11.2	111
La	9000	8750±55	137	8613	8887	0.63	97.2
Nd	7000	7230±97	241	6989	7471	1.34	103
Sm	1000	982±104	257	724	1240	10.6	98.2
Sc	40	45±3.5	8.7	36.3	53.7	7.78	112
Tb	100	109±13	32.3	76.7	141.3	11.9	109
Yb	200	187±12.8	31.8	155	218.8	6.85	93.5

Table S7. Initial concentrations (µg/kg) of REEs in spiked samples.

	UAE procedure		MAD procedure	
	Sample no. 9 (ground roasted coffee)	Sample no. 38 (instant coffee)	Sample no. 9 (ground roasted coffee)	Sample no. 38 (instant coffee)
Pr	0.331	0.526	0.501	0.768
Nd	1.06	0.413	1.57	0.612
Sm	1.59	0.773	0.232	0.570
Eu	2.50	1.18	2.58	1.26
Gd	1.64	0.645	0.456	0.496
Tb	0.075	0.077	0.051	0.056
Dy	0.134	0.353	0.194	0.516
Ho	0.061	0.084	0.097	0.126
Er	0.095	0.195	0.139	0.279
Tm	0.022	0.070	0.022	0.075
Yb	0.064	0.055	0.091	0.078
Lu	0.049	0.030	0.052	0.032
Sc	2.09	6.18	3.17	9.17
Y	1.36	1.56	1.67	1.88
Ce	2.44	1.61	4.04	2.60
La	1.46	0.846	2.1	1.17

Table S8a. Recovery (R) and precision (RSD) for spiking of UAE experiments (Spike 1: 0.5 µg/kg; Spike 2: 5.0 µg/kg).

Element	Ground roasted coffee (Sample ID: 9)						Instant coffee (Sample ID: 38)					
	Spike 1 (0.5 µg/kg)			Spike 2 (5.0 µg/kg)			Spike 1 (0.5 µg/kg)			Spike 2 (5.0 µg/kg)		
	Found µg/kg	RSD%	R%	Found µg/kg	RSD%	R%	Found µg/kg	RSD%	R%	Found µg/kg	RSD%	R%
Pr	0.775	5.45	88.7	5.18	10.7	96.9	0.980	8.04	90.6	5.36	7.34	96.6
Nd	1.47	7.44	82.1	6.19	8.20	103	0.868	10.7	91.0	5.43	11.2	100
Sm	2.00	8.35	81.1	6.30	6.83	94.1	1.23	13.0	92.2	5.99	10.2	104
Eu	2.91	2.44	83.1	7.61	7.41	102	1.61	9.08	86.4	6.24	8.27	101
Gd	2.10	5.74	92.4	7.04	2.24	108	1.11	6.76	93.3	5.56	9.10	98.4
Tb	0.577	7.60	100	5.00	8.30	98.5	0.568	11.5	98.2	4.98	5.17	98.1
Dy	0.632	10.9	99.6	4.97	7.38	96.8	0.811	11.5	91.5	4.93	5.08	91.5
Ho	0.572	11.1	102.2	4.47	6.78	88.2	0.544	5.56	92.1	4.39	7.46	86.2
Er	0.556	12.6	92.2	4.90	8.51	96.1	0.615	12.6	84.0	5.50	8.58	106
Tm	0.545	8.53	104	5.28	3.63	105	0.499	12.2	85.7	5.49	7.78	108
Yb	0.520	9.39	91.1	4.78	9.65	94.4	0.529	10.6	94.8	4.76	12.0	94.0
Lu	0.566	8.03	103	4.42	4.29	87.4	0.510	12.7	96.0	4.27	10.6	84.7
Sc	2.51	10.6	83.2	7.10	6.38	100	6.68	4.22	99.7	10.7	1.38	90.7
Y	1.83	6.03	92.8	5.83	5.29	89.2	2.01	6.34	91.3	6.60	9.60	101
Ce	2.92	2.06	95.8	7.21	7.36	95.4	2.02	6.25	82.0	6.52	5.36	98.3
La	1.98	8.25	104	6.24	9.02	95.6	1.25	11.7	80.1	5.68	9.86	96.6

Table S8b. Recovery (R) and precision (RSD) for spiking of UAE experiments (Spike 3: 40 µg/kg; Spike 4: 200 µg/kg).

Element	Ground roasted coffee (Sample ID: 9)						Instant coffee (Sample ID: 38)					
	Spike 3 (40 µg/kg)			Spike 4 (200 µg/kg)			Spike 3 (40 µg/kg)			Spike 4 (200 µg/kg)		
	Found µg/kg	RSD%	R%	Found µg/kg	RSD%	R%	Found µg/kg	RSD%	R%	Found µg/kg	RSD%	R%
Pr	40.1	11.8	99.5	204	2.04	102	38.4	8.45	94.6	193	6.09	96.5
Nd	41.5	7.95	101	176	4.59	87.5	38.4	8.60	94.9	209	2.61	104
Sm	40.1	5.67	96.4	176	5.96	87.0	41.0	4.92	101	202	5.00	101
Eu	38.4	6.56	89.8	193	6.45	95.0	39.4	9.47	95.7	194	3.71	96.3
Gd	38.9	6.53	93.2	200	7.40	99.3	40.7	10.4	100	181	6.61	90.1
Tb	38.0	9.99	94.9	198	9.58	98.9	37.5	2.10	93.5	210	3.88	105
Dy	39.9	9.48	99.3	206	5.57	103	38.7	8.05	95.9	198	9.72	98.6
Ho	39.7	6.27	99.0	200	7.61	99.9	43.1	6.98	107	189	8.82	94.4
Er	36.0	9.80	89.7	198	5.05	98.8	39.6	1.76	98.5	195	6.77	97.6
Tm	39.5	8.27	98.8	190	10.6	95.2	39.5	6.79	98.5	182	9.45	91.1
Yb	41.5	10.4	104	194	4.86	97.2	41.2	4.84	103	200	8.86	100
Lu	38.3	8.09	95.7	179	2.60	89.6	40.8	8.91	102	187	2.32	93.4
Sc	40.8	6.90	96.7	201	7.43	99.6	42.3	9.93	90.4	203	7.83	98.3
Y	41.0	9.13	99.0	198	8.63	98.5	37.6	2.15	90.1	202	8.64	100
Ce	39.4	5.22	92.3	199	6.81	98.2	41.2	8.02	99.0	206	4.68	102
La	39.7	8.77	95.6	201	8.65	99.5	41.0	8.58	100	200	9.50	99.5

Table S8c. Recovery (R) and precision (RSD) for spiking of MAD experiments (Spike 1: 0.5 µg/kg; Spike 2: 5.0 µg/kg).

Element	Ground roasted coffee (Sample ID: 9)						Instant coffee (Sample ID: 38)					
	Spike 1 (0.5 µg/kg)			Spike 2 (5.0 µg/kg)			Spike 1 (0.5 µg/kg)			Spike 2 (5.0 µg/kg)		
	Found µg/kg	RSD%	R%	Found µg/kg	RSD%	R%	Found µg/kg	RSD%	R%	Found µg/kg	RSD%	R%
Pr	0.906	6.42	80.9	5.57	10.8	101	1.29	10.7	104	5.82	8.50	101
Nd	2.08	8.27	102	6.66	10.6	102	1.03	11.1	83.9	5.04	5.87	88.5
Sm	0.701	7.99	93.8	5.55	5.53	106	1.04	12.1	93.6	5.13	12.2	91.1
Eu	3.03	8.57	89.9	7.18	11.5	92.0	1.68	4.67	83.6	6.33	9.09	101
Gd	1.02	7.77	113	4.99	11.2	90.7	1.02	8.19	105	5.27	8.73	95.5
Tb	0.460	11.3	81.7	4.69	5.81	92.9	0.564	8.76	101	4.67	7.61	92.2
Dy	0.629	8.36	87.0	5.29	7.62	102	1.07	6.67	111	5.71	7.15	104
Ho	0.557	11.3	92.0	4.62	4.40	90.5	0.602	11.0	95.1	4.79	10.2	93.2
Er	0.597	10.6	91.7	4.57	10.4	88.6	0.757	8.87	95.7	5.47	12.3	104
Tm	0.465	9.02	88.6	4.84	5.52	96.3	0.523	12.0	89.7	5.26	10.2	104
Yb	0.516	5.62	85.0	4.59	7.04	90.0	0.510	7.95	86.4	4.80	7.68	94.4
Lu	0.504	7.32	90.5	4.52	5.79	89.4	0.483	9.05	90.2	5.09	9.79	101
Sc	3.64	8.96	93.1	7.90	5.33	94.7	9.71	1.83	107	14.0	2.62	96.3
Y	2.09	10.0	84.0	6.38	8.22	94.2	2.42	9.29	107	6.77	9.76	97.7
Ce	4.51	5.60	93.5	8.86	9.45	96.4	3.00	11.4	79.3	7.86	5.03	105
La	2.64	9.75	109	6.52	10.1	88.3	1.62	12.2	90.1	5.86	6.11	93.7

Table S8d. Recovery (R) and precision (RSD) for spiking of MAD experiments (Spike 3: 40 µg/kg; Spike 4: 200 µg/kg).

Element	Ground roasted coffee (Sample ID: 9)						Instant coffee (Sample ID: 38)					
	Spike 3 (40 µg/kg)			Spike 4 (200 µg/kg)			Spike 3 (40 µg/kg)			Spike 4 (200 µg/kg)		
	Found µg/kg	RSD%	R%	Found µg/kg	RSD%	R%	Found µg/kg	RSD%	R%	Found µg/kg	RSD%	R%
Pr	38.2	4.78	94.4	193	6.23	96.0	37.8	7.78	92.5	193	3.19	96.2
Nd	39.2	10.2	94.0	204	1.70	101	40.6	10.4	100	202	3.20	101
Sm	38.7	9.09	96.3	197	6.80	98.4	35.6	7.51	87.5	199	2.18	99.1
Eu	38.5	8.22	89.7	197	3.48	97.2	42.1	8.29	102	203	6.14	101
Gd	37.4	5.97	92.5	179	3.22	89.4	39.8	6.57	98.2	195	5.60	97.4
Tb	41.5	9.08	104	197	8.26	98.5	41.4	9.28	103	202	5.03	101
Dy	40.8	4.74	101	199	8.95	99.5	39.9	9.58	98.3	204	6.43	101
Ho	41.3	6.74	103	198	3.68	98.9	38.0	8.89	94.7	201	5.10	100
Er	43.6	5.53	109	198	7.16	99.0	42.5	10.8	105	200	7.24	99.8
Tm	34.4	8.51	86.1	184	9.53	91.9	42.9	8.42	107	205	5.79	102
Yb	39.5	7.59	98.6	182	5.83	90.9	39.9	8.12	99.6	196	4.63	97.9
Lu	38.3	4.41	95.7	205	9.12	103	38.9	9.76	97.2	208	7.51	104
Sc	42.0	10.5	97.2	197	8.09	97.0	48.4	4.88	98.1	206	1.46	98.4
Y	40.4	10.7	96.9	202	8.00	99.9	38.1	4.89	90.5	200	6.58	99.0
Ce	41.1	7.34	92.6	194	6.26	95.2	41.7	9.73	97.6	203	6.73	100
La	42.8	10.7	102	199	3.25	98.4	42.9	9.65	104	205	8.13	102

Table S9. Descriptive statistics of the REE concentrations in coffee..

	Min (μg/kg)	Max (μg/kg)	Mean (μg/kg)	STD (μg/kg)	Skewness	Kurtosis
Pr	0.27	39.7	1.33	4.12	9.08	85.1
Nd	0.161	148	4.11	15.5	9.07	83.5
Sm	0.061	33.8	1.09	3.52	8.98	83.8
Eu	0.090	12.9	1.39	1.7	4.92	29.2
Gd	0.092	76.2	2.45	7.93	9.04	84.7
Tb	0.008	6.68	0.191	0.697	9.09	85.2
Dy	0.032	31.3	0.74	3.26	9.28	87.7
Ho	0.012	5.67	0.161	0.593	9.12	85.5
Er	0.002	16.15	0.438	1.68	9.16	86.0
Tm	0.002	1.83	0.063	0.191	8.99	84.0
Yb	0.010	9.74	0.286	1.02	8.93	83.0
Lu	0.001	1.42	0.066	0.151	8.24	74.0
Sc	2.09	14.5	4.68	1.99	2.01	6.32
Y	0.313	169	4.43	17.6	9.24	87.2
Ce	0.815	347	11.7	36.4	8.82	81.9
La	0.366	186	5.96	19.4	9.01	84.3

Table S10. Pearson correlation matrix for REEs in coffee samples.

	Pr	Nd	Sm	Eu	Gd	Tb	Dy	Ho	Er	Tm	Yb	Lu	Sc	Y	Ce
Nd	**0.863**														
Sm	**0.876**	**0.920**													
Eu	**0.647**	**0.642**	**0.706**												
Gd	**0.906**	**0.974**	**0.938**	**0.666**											
Tb	**0.868**	**0.870**	**0.889**	**0.693**	**0.905**										
Dy	**0.836**	**0.856**	**0.838**	**0.566**	**0.868**	**0.878**									
Ho	**0.770**	**0.746**	**0.763**	**0.518**	**0.762**	**0.862**	**0.854**								
Er	**0.735**	**0.729**	**0.751**	0.440	**0.741**	**0.805**	**0.838**	**0.862**							
Tm	**0.606**	**0.525**	**0.583**	0.450	**0.589**	**0.701**	**0.668**	**0.734**	**0.677**						
Yb	**0.543**	**0.566**	**0.550**	0.106	**0.558**	**0.581**	**0.658**	**0.697**	**0.754**	**0.582**					
Lu	0.409	0.342	0.397	0.155	0.369	**0.516**	**0.556**	**0.624**	**0.650**	**0.648**	**0.680**				
Sc	0.018	-0.247	-0.172	-0.218	-0.208	-0.036	0.026	0.121	0.122	0.108	0.262	0.382			
Y	**0.835**	**0.857**	**0.861**	**0.593**	**0.860**	**0.876**	**0.923**	**0.893**	**0.893**	**0.694**	**0.753**	**0.601**	0.072		
Ce	**0.879**	**0.976**	**0.914**	**0.637**	**0.980**	**0.860**	**0.823**	**0.707**	**0.694**	**0.532**	**0.528**	0.319	-0.260	**0.804**	
La	**0.851**	**0.972**	**0.905**	**0.677**	**0.957**	**0.835**	**0.792**	**0.687**	**0.691**	0.497	**0.527**	0.311	-0.266	**0.812**	**0.971**

Table S11. Principal component analysis showing two extracted components with eigenvalues greater than one, and their loadings.

Variable	PC1	PC2
Pr	0.27391	-0.06276
Nd	0.28099	-0.18626
Sm	0.28051	-0.14282
Eu	0.19437	-0.26438
Gd	0.28517	-0.17146
Tb	0.28581	-0.02688
Dy	0.28095	0.04742
Ho	0.27076	0.17398
Er	0.26061	0.18419
Tm	0.21105	0.24063
Yb	0.20584	0.33149
Lu	0.16627	0.46921
Sc	0.00325	0.53333
Y	0.28665	0.09769
Ce	0.27512	-0.2101
La	0.27193	-0.22009
Eigenvalue	11.0265	2.34858
Variance,%	68.92	14.68
Cumulative,%	68.92	83.59
